# Subjective Discomfort during Botulinumtoxin Injections Dependent on Injection Site and Needle Size: A Comparison Between 30G, 33G and 34G Needles

**DOI:** 10.1007/s00266-024-03877-7

**Published:** 2024-03-05

**Authors:** Till A. Kämmerer, Randolf Bertlich, Daniela Hartmann, Mark Jakob, Bernhard G. Weiss, Ines Bertlich, Friedrich Ihler, Paul Severin Wiggenhauser, Mattis Bertlich

**Affiliations:** 1 Department of Dermatology and Allergy, LMU University Hospital, LMU Munich, Frauenlobstr. 9-11, 80337 Munich, Federal Republic of Germany; 2 Dermafit Institute for Cosmetic Dermatology, Hervester Str. 55, 45768 Marl, Federal Republic of Germany; 3 Department of Otorhinolaryngology, Head and Neck Surgery, Ludwig-Maximilians University of Munich, Marchioninistr. 15, 81377 Munich, Federal Republic of Germany; 4 Department of Dermatology, Ruprecht-Karls-University of Heidelberg, Im Neuenheimer Feld 440, 69120 Heidelberg, Federal Republic of Germany; 5 Department of Otorhinolarnygology, Head and Neck Surgery, Greifswald University Medicine, Fleischmannstraße 8, 17475 Greifswald, Federal Republic of Germany; 6 Department of Hand and Plastic Surgery, LMU University Hospital, Ziemsenstr. 5, 80336 Munich, Federal Republic of Germany

**Keywords:** Botulinumtoxin, Needle size, Pain, Discomfort

## Abstract

**Background:**

Botulinumtoxin application in the face is amongst the most common aesthetic procedures in the head and neck region. It also has numerous medical uses. One of the main reasons for patients to refrain from it is the subjective discomfort that is experienced during injections.

**Objectives:**

The study at hand aimed to determine whether needles with 33G and 34G offer an advantage in terms of individual pain perception during botulinumtoxin injections.

**Methods:**

We conducted a prospective study where patients were asked to grade subjective discomfort on a visual analogue scale for each region (forehead, glabella, temple) that was treated directly after treatment and 15 minutes after. Patients were treated with 30G, 33G or 34G needles, respectively.

**Results:**

Ninety-nine patients that underwent treatment of 189 regions were included in the study. Patients were evenly distributed amongst the different needle sizes and regions. Subjective discomfort was greatest in all regions for 30G needles (3.9  ± 1.6 forehead, 4.3 ± 1.7 glabella and 4.0 ± 1.6 temple) followed by 33G (2.7 ± 1.5 forehead, 2.7 ± 1.9 glabella and 2.2 ± 1.2 temple) and 34G (1.7 ± 1.2 forehead, 1.6 ± 1.4 glabella and 1.6 ± 1.4 temple). All differences between needle size were statistically significant (*p* < 0.05)

**Conclusion:**

33G and 34G needles seem to offer smaller discomfort during BTX treatments of the head and neck, with 34G being superior to 33G.

**Level of Evidence III:**

This journal requires that authors assign a level of evidence to each article. For a full description of these Evidence-Based Medicine ratings, please refer to the Table of Contents or the online Instructions to Authors www.springer.com/00266

## Introduction

Local treatments with botulinumtoxin (BTX) are amongst the most common non-surgical interventions in the head and neck. Injections of BTX are effective and are licensed for reducing wrinkles of the forehead, the glabella and the temple (“crow’s feet”). Moreover, BTX injections also show great efficacy in numerous medical therapies, including (but not limited to) tension headaches [1], migraines [2] and Frey’s syndrome [3] or salivary fistula [4] after parotid surgery. However, BTX has to be delivered by transcutaneous injections. Despite being safe and efficient in all of the above-mentioned indication, patients regularly report that discomfort during injections is amongst the most common downsides of treatment.

Consequently, numerous techniques have been used to mitigate the discomfort of injections. It has been shown that repeated use of needles decreases the respective sharpness and injections require more physical force [5]. Consequently, repeated injections with the same canula are bound to be more painful. Moreover, increased canula size has been suggested to decrease discomfort, as a needle with a smaller diameter are bound to injure a smaller area of the skin.

However, the scientific literature on this matter is conflicting. Moreover, most studies compared needles regularly used for BTX injections (commonly 27G or 30G) with needles of a smaller diameter (commonly 32G). Nevertheless, several companies have developed considerably smaller needles in the past few years with 33G and even 34G. As there is no scientific evidence as to whether these needle sizes offer an advantage in terms of subjective discomfort, we conducted a prospective cohort study to determine the subjective discomfort dependent on the needle size used during BTX injections.

## Materials and Methods

The study at hand was registered with the appropriate authorities (ethics committee of the University A) under the file no. 03-0524/KB. All patients gave informed consent to participate in the study. The entire investigation was performed according to the rules laid out by the Declaration of Helsinki.

Only patients aged 18 years or older that underwent BTX injections for facial wrinkles were included in the study. Only patients that had previously undergone BTX injections with 30G needles and did not report them as exceedingly painful were included in the study. Injections were performed in outpatient settings in the respective departments (Department of Dermatology and Allergy, University A, Department of Otorhinolaryngology, Head and Neck Surgery, University A, Department of Hand and Plastic Surgery, University A, Department of Dermatology, University B and two private practices).

Data collection started as early as 1 June 2023 and finished 1 September 2023.

All physicians had previously used 30G (BD Insulin Syringes Ultra-Fine, 8mm×30G needle, Becton Dickinson, Franklin Lakes, New Jersey, USA) syringes for BTX injections. 30G low dead space needles provided by the authors were therefore used as reference (30G Low Dead Space Hub, 30G×13mm). The 30G as well as 33G (Low Dead Spaces Hub 33G×13mm Needle) and 34G (The Invisible Needle®, 34G×13mm) needles was purchased from the same manufacturer (all from TSK Laboratory Europe B.V., Oisterwijk, the Netherlands) and offered as alternatives to the 30G standard needles. 32G needles were purchased from the same manufacturer, but due to differences in the way they were made (only 4 and 6mm needles were available) not deemed suitable for the study at hand. Patients were informed that they received either a standard or a different, smaller needle and were asked to rate the individual discomfort after the treatment of each region. Needles were disposed after the treatment of one region and a new one was used for the next region. Physicians performing the injections recorded data on the number of injections and the number of AE that were injected in each region. Selection of needle size was independent of the patients and subject to the individual physician.

Only patients that underwent treatment with BOCOUTURE®, XEOMIN® (Incobotulinumtoxin A), VISTABEL® or BOTOX® (Onabotulinumtoxin A) were included in the study, as these preparations are all measured in Allergan Units (AU). All preparations were dissolved according to the manufacturers recommendations (1.25ml per 50 AU or 2.50ml per 100 units, resulting in 4 AU per 100µl).

Patients were asked to grade the individual pain of the treatment for each region (forehead, glabella, temple) directly after the treatment and 15 minutes after treatment on a visual analogue scale (VAS). Patients were offered a straight line of 10cm and were asked to indicate their individual discomfort with a mark somewhere between 0cm (minimum discomfort) and 10cm (maximum discomfort). Afterwards, the distance between minimum discomfort and the mark was measured. This approach is recommended by national guidelines and has been scientifically validated [6]. After the second grading, the physician examined the patients whether any sign of hematoma had arisen in the areas that had been treated.

Statistical analysis was carried out using project R for Mac (Build 4.0.2 for Mac, The R Project for Statistical Computing, http://www.r-project.org/). To detect significant differences between individual groups, Student’s t-test was used. Where other tests were used, these are indicated during the manuscript. A p-value smaller than 0.05 was considered significant.

## Results

Overall, 99 patients were included in the study, in which 189 regions were treated. Eighty patients were female and 19 patients were male. Average age was 42.9 ± 12.6 years, with the youngest patient being 23 years and the oldest patient being 80 years old. Patients were evenly distributed amongst 30G, 33G and 34G needles (32, 32 and 35 patients, respectively) and regions (65, 86 and 38 patients, respectively). General characteristics and individual distributions for each group can be found in table 1. *Χ*^2^ test did not reveal a significant imbalance in terms of distributions amongst the different groups or regions (*p* = 0.736).

**Table 1:** General distribution of patients amongst needle sizes and regions

	General characteristics			No. of treatments		
	n	Sex (f/m)	Age (yrs)	Forehead	Glabella	Temple
Overall	99	80 (80.8%)/19 (19.2%)	42.9 ± 12.6 [23 − 80]	65	86	38
30G	32	24 (75.9%)/8 (25.0%)	45.8 ± 14.7 [24 − 80]	21	29	15
33G	32	25 (78.1%)/7 (21.9%)	43.3 ± 11.0 [23 − 64]	18	26	13
34G	35	32 (91.4%)/3 (8.6%)	40.1 ± 11.4 [27 − 76]	26	31	10

Values are median ± standard deviation. Brackets indicate minimum and maximum values

The number of injections as well as the number of AU was similar amongst each region and independent of needle size, as proven by *Χ*^2^ test (*p*=0.649 and *p*=0.816, respectively).

The exact values can be found in table 2.

**Table 2:** Average number of injections per region and average amount of AU applied per region

	Forehead	Glabella	Temple
No. of injections per region			
Overall	8.1 ± 2.6	5.1 ± 0.8	5.8 ± 0.6
30G	8.5 ± 2.5	5.2 ± 0.8	6.0 ± 0.4
33G	6.5 ± 1.9	5.0 ± 0.8	5.7 ± 0.7
34G	8.8 ± 2.7	5.2 ± 0.8	5.8 ± 0.6
Amount (IE) of BTX applied per region			
Overall	15.2 ± 4.3	14.4 ± 3.2	11.6 ± 2.6
30G	17.4 ± 4.9	14.9 ± 3.1	12.2 ± 2.9
33G	13.3 ± 3.5	12.8 ± 3.1	11.1 ± 2.0
34G	14.7 ± 3.5	15.4 ± 3.5	11.6 ± 2.9

**Fig 1.**
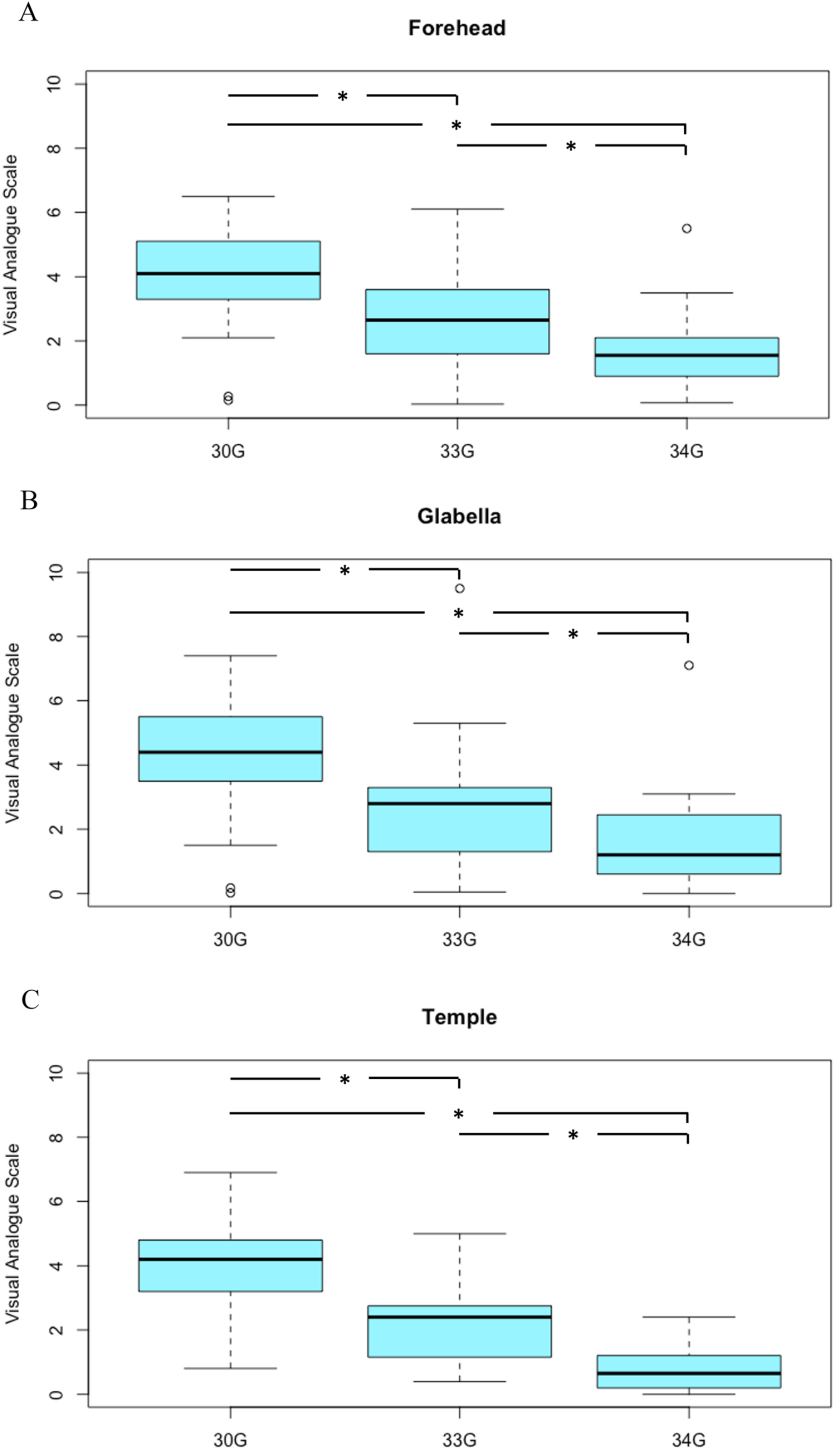
Averages of VAS directly after treatment of each location dependent on needle size. * = *p* < 0.05

Visual analogue scale directly after treatment of the forehead with 30G needles showed an average discomfort of 3.9 ± 1.6, as reported on the visual analogue scale. This was significantly greater than the discomfort reported in 33G (2.7 ± 1.5, *p*=0.027) and 34G (1.7 ± 1.2, *p*<0.001). The difference between 33G and 34G was also statistically significant (*p*=0.023). Treatments of the glabella showed an average of 4.3 ± 1.7 on the visual analogue scale for 30G needles. This was significantly greater than the discomfort reported for 33G (2.7 ± 1.9, *p*=0.003) and 34G needles (1.6 ± 1.4, *p*<0.001). The difference between 33G and 34G was also statistically significant (*p*=0.013). Temple treatments were numerically lower, however, evenly distributed amongst individual groups. 30G treatments averaged a VAS of 4.0 ± 1.6, which was significantly higher than with 33G needles (2.2 ± 1.6, *p*=0.002). 34G yielded an even smaller VAS score (0.9 ± 0.8) which was significantly lower than 30G (*p*<0.001) and 33g (*p*=0.009). A graphical representation can be found in Fig. 1.

**Fig 2.**
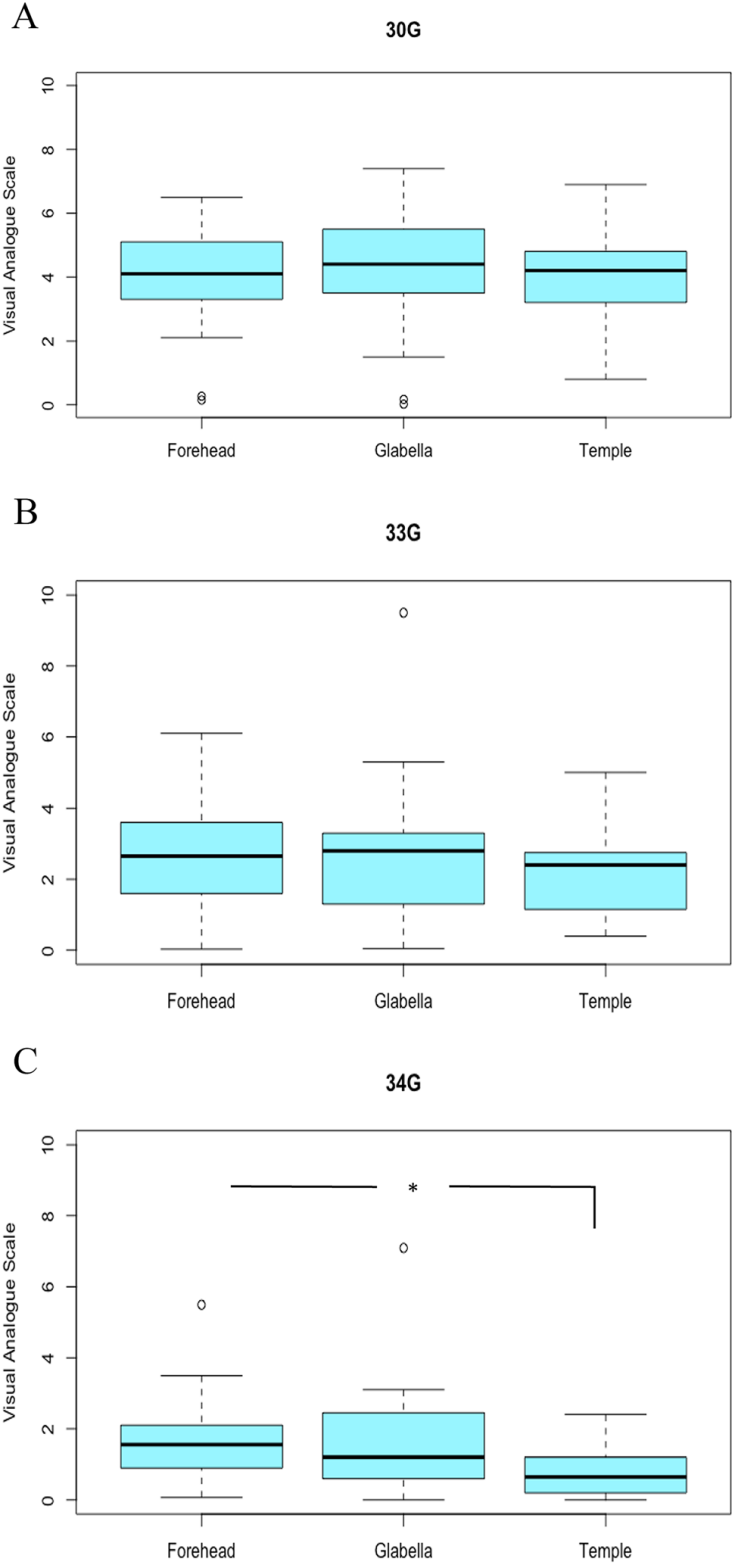
Average of VAS directly after treatment of each location for needle size, dependent on location. * = *p* < 0.05

Comparisons amongst injection sites yielded very few significant results. Amongst 30G needles, there were no significant differences between the forehead and the glabella (*p*=0.459), between the forehead and the temple (*p*=0.863) and the glabella and the temple (*p*=0.614). Amongst the 33G needles, there were no statistically significant differences between the forehead and the glabella (*p*=0.962), the forehead and the temple (*p*=0.295) or the glabella and the temple (*p*=288). In the patients that were treated with 34G needles, forehead and glabella showed no significant differences (*p*=0.699) as well as glabella and temple (*p*=0.078). However, the VAS between forehead and temple was statistically significant (*p*=0.031). Visual representations can be found in Fig. 2.

**Fig 3.**
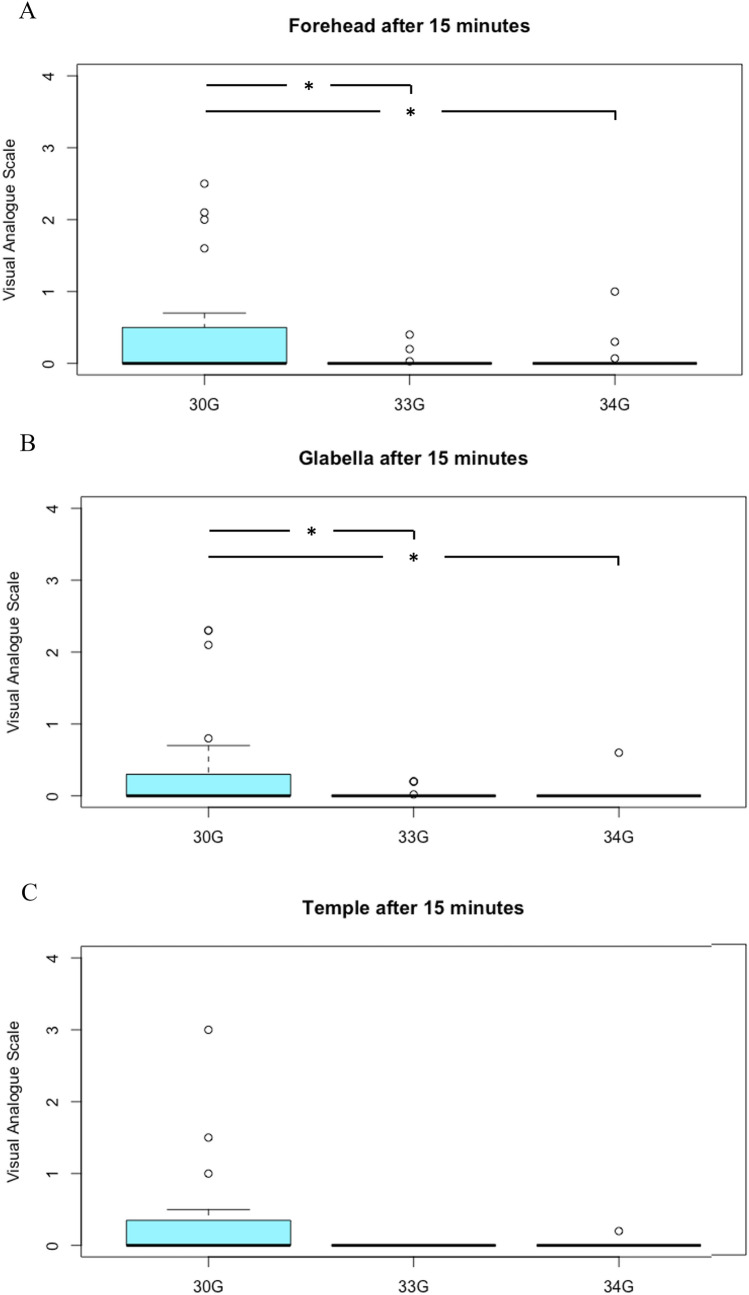
Average of VAS 15 minutes after treatment of each location, dependent on needle size. * = *p* < 0.05

In terms of the measurements after 15 minutes, all patients reported little discomfort. In the group that was treated with 30G needles, average VAS for the forehead was 0.5 ± 0.8, after 15 minutes, while the group treated with 33G needles indicated a VAS of 0.0 ± 0.1 and the 34G group indicated 0.1 ± 0.2. Both the VAS for 33G (*p*=0.019) and 34G (*p*=0.025) were significantly smaller than the VAS indicated for 30G. The difference between 33G and 34G was not statistically significant (*p*=0.705). In terms of the glabella, VAS for 30G was 0.3 ± 0.7, 33G VAS was 0.0 ± 0.1 and 34G VAS was 0.0 ± 0.1. Again, both 33G (*p*=0.019) and 34G (*p*=0.018) showed a significant difference compared to 30G, while there was no significant difference between the two groups (*p*=0.847). Similar observations were made in the temple region, where the 30G group indicated a VAS of 0.4 ± 0.8, while the 33G group indicated a VAS of 0.0 ± 0.0 and the 34G group a VAS of 0.0 ± 0.1. Unlike in the forehead and the glabella, neither 33G (*p*=0.078) nor 34G (*p*=0.094) showed a significant difference to 30G needles. The difference between 33G and 34G was also not statistically significant (*p*=0.343). Visual representations can be found in Fig. 3.

Thirteen Patients showed hematoma after treatments with 30G needles, while one patient showed a hematoma after treatment with 33G needles and two patients showed hematoma after treatments with 34G needles. The difference between these groups was statistically significant (*p* = 0.001, *Χ*^2^-test).

## Discussion

As facial application of BTX is a very common procedure that is used for numerous aesthetic and medical indications, there are numerous studies that have attempted to reduce the discomfort during injection by various techniques [7–9]. Unsurprisingly, there are also several studies that have tried to assess the discomfort of BTX injections dependent on needle size [10–13]. A brief overview of these studies can be found in table 3.

**Table 3:** Overview of the previous studies that assessed needle size and subjective discomfort during BTX treatments

Study	Cannulas compared	No. of injections and injection sites	Key findings
Yoomtoob et al., Ophthalmic plastic and reconstructive surgery, 2009	30G and 32G	30 patients, periocular, one side treated with 30G and contralateral with 32G	No significant differences between cannula size
Price et al., Dermatologic surgery, 2010	30G and 32G	37 patients in the head and neck	No significant differences between cannula size
Skiveren et al., Acta dermato-venerologica, 2011	27G and 30G	38 patients treated for axillary hyperhidrosis	Significantly more discomfort in 27G needles in specific patient subsets
Alam et al., JAMA Dermatology, 2015	30G and 32G	20 patients in head and neck/forearm	Significantly more discomfort in the 30G group

Interestingly, the findings amongst these studies are not homogenous. Only one of the four studies that assessed the discomfort of BTX injections found a significant difference between 30G and 32G needles. Nonetheless, the study at hand is very much in line with the latter study as the number of patients that reported a considerable discomfort (VAS > 5) while undergoing 30G injections was quite similar in the study at hand (34.4%) and in the respective study (40.0%).

However, there are some very major differences compared with the study at hand. Firstly, this is—to the best of our knowledge—the first time that the subjective discomfort of 33G and 34G needles has been recorded in a scientific study. Moreover, the collective at hand is considerably larger than the previously mentioned studies. This is relevant as all of these studies found a difference between subjective VAS scales in terms of BTX discomfort—yet most of the differences were not statistically significant. Subsequently, these studies might have been just statistically underpowered.

Interestingly enough, Arendt-Nielsen and colleagues conducted a study in 2006 where different needle sizes (23G–32G) were compared in a device that always perforated the participants skin with the same force, velocity and angle. In this study, they were able to demonstrate a clear relationship between needle size and discomfort of injections. Taking this into account, it may very well be possible that individual physicians handling is also a key component in discomfort during BTX injections, causing the inhomogeneous set of results that have been published to date. Fittingly, there are more scientific reports about the correlation of needle size and subjective discomfort that further support this view, yet did not address BTX injections [14]. Moreover, this view is even further supported by the fact that almost 1/3 of all patients treated with 30G needles showed signs of hematoma. While this could partially account for the higher amount of pain that has been indicated with 30G needles (as patients with hematomas tend to show increased pain after injection) [15], the overall rate seems somewhat excessive and may be—in our opinion—subject to individual physician treatments. Another explanation might be a different angle and/or cut of the needle tip between 30G needles used in the study at hand and other commercially available appliances. The extended tendency for bruising might also explain the fact that 30G needles seem to be more painful even 15 minutes after the injection, even though the vast majority of patients indicated little to no discomfort at all at that point in time.

However, despite these considerations, we found strong evidence that injections of BoNT with 34G and 33G needles cause significantly less discomfort than those performed with 30G needles, with a significantly smaller risk of hematoma. In terms of 34G and 33G needles, 34G seems to cause even smaller discomfort.

Still, other factors also need to be taken into account, firstly, BD Insulin Syringes (which were regularly used by all participating physicians prior to this study), while coming at a considerably lower price (0,23€ incl. VAT per Syringe). 33G and 34G needles that were used in the study at hand in contrast had—even though both were designed for smallest possible dead space—still a macroscopically visible (albeit small) dead space. Moreover, the needles came at a considerably higher price (0,55€ excl. VAT for 33G and 0,65€ excl. VAT for 34G). Hence, the fact that the majority of physicians in this study had previously used 30G insulin syringes been potentially rather an economical question than one of patient discomfort.

Finally, the limitations of the study at hand need to be addressed. Firstly, 32G needles with similar length and cut were not available as controls that have been scientifically validated. Moreover, owed to the number of treating physicians, individual treatment strategies might have influenced individual outcomes, as it has been shown that communication with the patient may significantly impact individual pain perception [16]. Furthermore, different cuts from different providers were not compared in the study at hand, as the cut of a needle might have considerable impact on individual pain perception. Another aspect that has not been addressed in this study was individual treatment strategies to minimize discomfort like pre-injection cooling [17] or vibration devices [18]. In addition, the study at hand lacks internal controls to correct for individuals who may be more prone to subjective pain. Moreover, individual physicians handling might have influenced the subjective perception of pain independent of needle size. This might particularly limit the individual perceptions in 33G and 34G needles, as the differences are—albeit statistically significant—numerically low. However, it is our conviction that the large number of participants outweighs this potential bias, as is supported by the clear results we were able to present.

## Conclusion

33G and 34G needles both offer significantly smaller subjective discomfort for the patient during BTX treatment in the head and neck. Moreover, both offer a significantly smaller risk for hematoma. 34G seems to be superior to 33G needles in terms of subjective discomfort.

## Data Availability

The original, anonymous dataset is available on reasonable request from the corresponding author. The authors of this manuscript declare that the data at hand have not been published, submitted or used in any other manuscript elsewhere.

## Abbreviations

AU: Allergan Units
BTX: Botulinumtoxin
VAS: Visual Analogue Scale
